# Performance of single and multi-atlas based automated landmarking methods compared to expert annotations in volumetric microCT datasets of mouse mandibles

**DOI:** 10.1186/s12983-015-0127-8

**Published:** 2015-12-01

**Authors:** Ryan Young, A. Murat Maga

**Affiliations:** Division of Craniofacial Medicine, Department of Pediatrics, University of Washington, Seattle, WA USA; Center for Developmental Biology and Regenerative Medicine, Seattle Children’s Research Institute, 1900 Ninth Ave, 98101 Seattle, WA USA; Department of Oral Biology, University of Washington, Seattle, WA USA

**Keywords:** Automated landmarking, Mandible, Geometric morphometrics, Multi-atlas segmentation, microCT

## Abstract

**Background:**

Here we present an application of advanced registration and atlas building framework DRAMMS to the automated annotation of mouse mandibles through a series of tests using single and multi-atlas segmentation paradigms and compare the outcomes to the current gold standard, manual annotation.

**Results:**

Our results showed multi-atlas annotation procedure yields landmark precisions within the human observer error range. The mean shape estimates from gold standard and multi-atlas annotation procedure were statistically indistinguishable for both Euclidean Distance Matrix Analysis (mean form matrix) and Generalized Procrustes Analysis (Goodall F-test). Further research needs to be done to validate the consistency of variance-covariance matrix estimates from both methods with larger sample sizes.

**Conclusion:**

Multi-atlas annotation procedure shows promise as a framework to facilitate truly high-throughput phenomic analyses by channeling investigators efforts to annotate only a small portion of their datasets.

**Electronic supplementary material:**

The online version of this article (doi:10.1186/s12983-015-0127-8) contains supplementary material, which is available to authorized users.

## Background

The growing use of high resolution three-dimensional imaging, such as micro-computed tomography (microCT), along with advances in visualization and analytic software have provided researchers with the opportunity to study the morphology of organisms in more detail. More and more researchers are turning to morphometric measurements obtained on 3D scans to quantitatively assess morphological differences in their experimental studies which might focus on effects of teratogens or mutations on craniofacial (CF) development, or simply study the normal CF development and variation [1–9]. In this context, geometric morphometric methods (GMM) are a suite of analytic techniques aimed at studying shape variation through annotation of landmarks corresponding to anatomical structures of interest, traditionally done by an expert [10, 11].

Thanks to the increasing accessibility of microCT scanning in general and, tissue staining protocols for microCT in particular, it is now possible to image dozens of adult mouse skulls or mandibles, or possibly hundreds of mice embryos in a single day. Yet, manual annotation of 3D datasets remains a labor-intensive process, requiring investigators to be trained on the accurate and consistent identification of anatomical landmarks. Manual annotation of the specimens imaged in a single work day can take much longer, perhaps as much as a few weeks. Furthermore, manual landmarking can introduce inter and intra-investigator error that can significantly impede the detection of subtle, yet biologically significant differences [8]. These differences are typically dealt with by obtaining multiple sets of annotations from the same specimen, possibly by multiple investigators, further delaying the process. Yet, the real power of quantitative morphometrics, and specifically geometric morphometrics, is its potential to detect slight shape changes so that researcher can study subtle within population variations or small-effect genetic variations. This, however, requires large sample sizes, and also more of the investigators efforts to be spent on data collection. The multivariate nature of GM analyses integrates very well with breeding experiments that manipulate the phenotype and help us identify genomic locus that are responsible for these complex phenotypes [4, 12–15]. These mapping studies have shown that the shape of a complex trait (such as a skull or mandible) is a highly polygenic trait, and the identification of the variants usually requires a large sample size. Thus, there is a strong need for high-throughput automated annotation of anatomical landmarks that will match the high-throughput imaging available.

There has been progress to speed up the annotation process, especially for 3D surface meshes that are acquired from surface scanners. Computer vision methods have been applied to automatically discriminate or, classify certain syndromes with various success [16–18]. These methods, typically based on machine learning algorithms, are better suited to classification problems and generally do not provide the shape variation information in an interpretable form. Other automatic approaches use mathematical and geometric criteria to define the landmarks [19, 20]. The benefit of fully automated landmarking using geometric criteria is its compatibility with the existing GMM analytic procedures. But, perhaps more significant is its promise of total elimination of any kind of observer variation as the algorithms are deterministic. Unfortunately, this require making series of assumptions in geometric arrangements of the anatomical features of interest, which may not be easily generalized to new annotation tasks, especially when different taxa or anatomical systems are considered. There are also landmark free approaches to quantify the shape variation [21, 22] in 3D. However, the statistical framework to analyze these kinds of data needs to be further developed and validated. With all its caveats regarding the speed and potential for various kinds of observer error, when executed carefully manual annotation of landmarks still remains as a robust and flexible procedure than can be extended to any anatomical system from any species with well-defined anatomical structures.

In this study our goal is to explore a methodology that leverages advanced image registration methods, which are commonly used in neuroimaging, into a flexible framework that channels investigators efforts into creating well-annotated, repeatedly verified set of ‘templates’ of landmarks that will serve as references to landmark a much larger study population. Our approach is conceptually similar to some previous attempts such as the atlas based classification of dysmorphologies in Fgfr2C342Y mutation [23] or the more recently published TINA toolkit [24]. In this context atlas (or template) is a dataset that serves as a reference to process the new (target) samples. Depending on the application atlas can be a single well characterized sample, or it can be constructed from a population (e.g. averaging all samples). In this study, we first look at the sensitivity of the atlas construction process (i.e. whether there is any bias in the outcome depending on from which sample the process initiated from), then compare single vs multi-atlas based automatic landmarking processes and evaluate them against manual annotation, which we refer to as ‘Gold Standard’ (GS). Lastly, we evaluate these findings in context of typical geometric morphometric analyses in which we compare the estimates of mean shape and shape variance and their implications.

## Results

### Atlas sensitivity to initial sample

To measure whether the initializing sample causes any bias in the outcome of the final atlas constructed, we tested for different outcomes by choosing unique initiating samples. The dice statistic and the correlation coefficient were used to evaluate the similarity of the atlases. For this study population, DRAMMS performs well and the outcome of the atlas was not dependent on the initializing sample (Table 1). This has been documented in other study populations such as neonatal and pediatric atlas construction [25].

**Table 1:** Dice (upper triangle) similarity scores and correlation coefficients (lower triangle) between atlases build from different initializing samples

	Sample 1	Sample 2	Sample 3	Sample 4
Sample 1		0.997	0.997	0.997
Sample 2	0.999		0.998	0.997
Sample 3	0.999	0.999		0.997
Sample 4	0.999	0.999	0.999	

DICE similarity is calculated as the ratio of twice the intersection of two images divided by sum the two images, with score of 1 representing two identical images. Four samples randomly chosen from the study population to initiate the atlas building process

### Surface selection to annotate single atlas

**Fig. 1:**
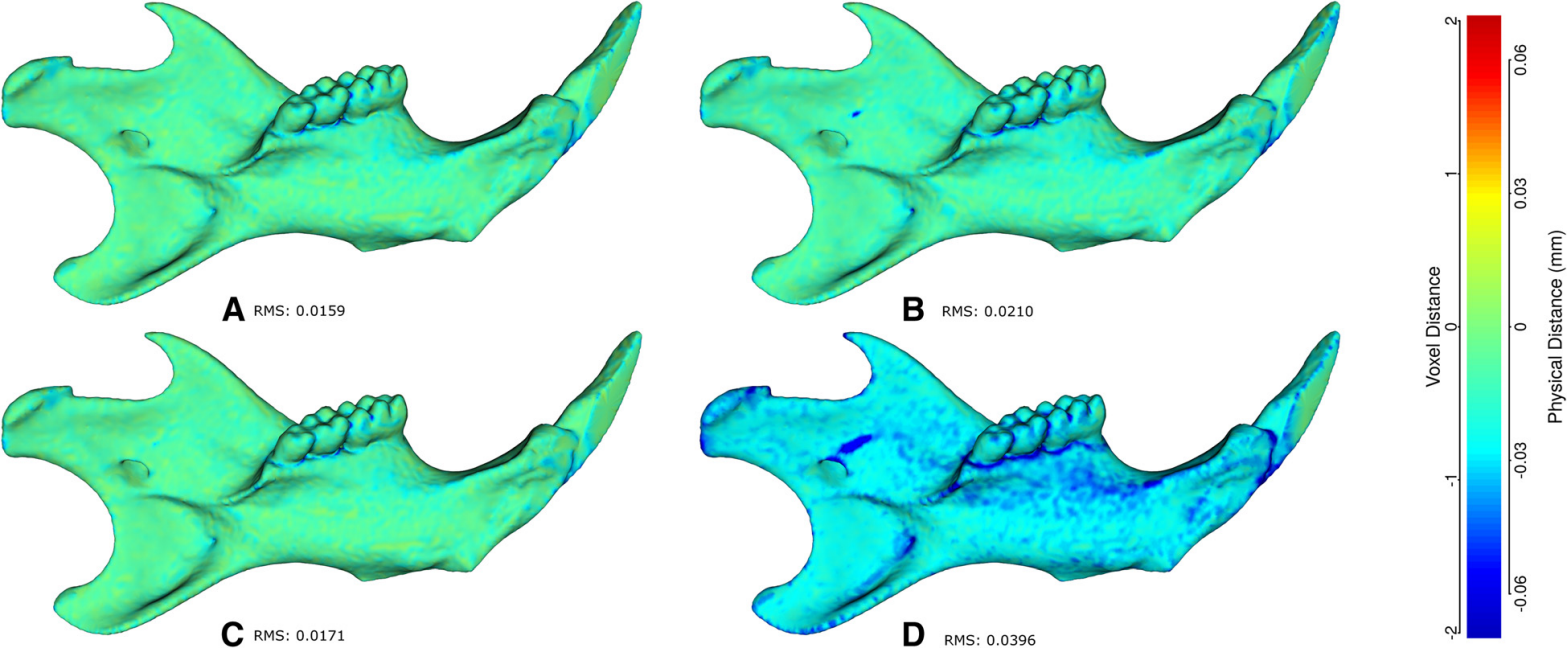
Visualization of the distances between the atlas surface that was landmarked (p90) and four other surfaces constructed. **a** 50 % Probability surface (p50); **b** 70 % Probability surface (p70); **c** Surface thresholded at grayscale value of 35. **d** Surface thresholded at grayscale value of 55. RMS: Root mean square error

Because our atlas is a constructed dataset, grayscale values of the voxels do not necessarily correspond to the density of the mandibular bone. Thus, a simple grayscale threshold may not consistently represent bone or tissue values as it does in a single microCT image. Therefore, we preferred to use a probability-based approach to choose our surface. Each sample was converted to a binary image and a probabilistic atlas was created such that the value of a voxel represented the frequency of that voxel being present in the population. We tested how the 90 % probability (p90) surface compared to two other probability surfaces (p70 and p50) and two other atlases based on different threshold settings and calculated the root mean square error between those and the p90. As shown in Fig. 1, the difference in most cases was minimal (less than one voxel), and we proceeded to landmarking using only the p90 surface.

### Comparison of automated landmarking results

**Fig. 2:**
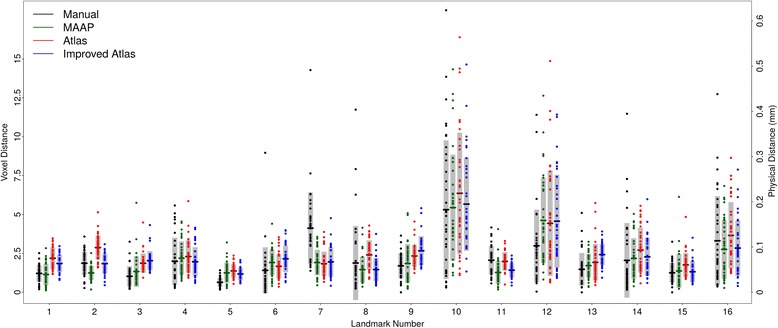
Comparison of automated landmarking methods to the gold standard. Each point is the digitization error associated with that landmark in one sample in a given method. Horizontal tick marks are means for each landmark. Gray bars indicate +/−1 SD from the mean

Difference between estimated landmarks *versus* the GS for all three methods in Euclidean distance is given in Fig. 2. Summary statistics on the observed landmark annotation errors are provided in Table 2. Single atlas annotation performed poorly. Using multiple samples to estimate landmark locations on the atlas did improve the mean errors, but multi-atlas method outperformed both. This is clearly demonstrated by a paired Mann–Whitney *U* test to assess the statistical significance (Table 2). We compared the linear distance between both sets of manual landmarks to the distance between the averaged landmarks (GS) and the automated method in question. For multi-atlas annotation method (MAAP) method, only two LMs out of the 16 had errors that exceeded that of GS (Table 2), whereas single and improved atlas had six.

**Table 2:** Digitization errors associated with each annotation technique.

LMs	1	2	3	4	5	6	7	8	9	10	11	12	13	14	15	16
Gold Standard	0.04	0.06	0.04	0.07	0.02	0.05	0.14	0.06	0.06	0.18	0.07	0.1	0.05	0.07	0.04	0.11
	0.02	0.02	0.02	0.05	0.01	0.05	0.08	0.08	0.03	0.15	0.03	0.08	0.04	0.08	0.02	0.10
Single Atlas	0.07*	0.10*	0.06*	0.08	0.05*	0.06	0.06^a^	0.08*	0.08*	0.22	0.07	0.15	0.07	0.09	0.06*	0.12
	0.02	0.03	0.02	0.04	0.01	0.03	0.03	0.03	0.02	0.13	0.02	0.11	0.04	0.05	0.03	0.07
Improved Atlas	0.06*	0.06	0.07*	0.07	0.04*	0.07*	0.07^a^	0.05	0.09*	0.19	0.05^a^	0.16	0.08*	0.08	0.04	0.10
	0.02	0.02	0.02	0.03	0.01	0.03	0.03	0.02	0.03	0.1	0.02	0.1	0.02	0.04	0.02	0.06
Multi Atlas	0.04	0.04^a^	0.05	0.07	0.04*	0.07*	0.06^a^	0.05	0.06	0.18	0.04^a^	0.16	0.06	0.07	0.05	0.09
	0.02	0.02	0.03	0.04	0.02	0.03	0.03	0.03	0.04	0.12	0.03	0.09	0.03	0.04	0.04	0.06

Mean (upper row) and standard deviations (lower row). Units are millimeters. A Paired Mann-Whitney *U* test was used to test for differences in digitization errors in each automated method with respect to gold standard at p=0.01. * indicates errors greater than the GS landmarks, while ^a^ denotes less. This is determined by a U statistic found in the tail. Error distributions indistinguishable from the GS landmark, which means U statistics not found in the tails, are not marked. *N* = 36 for all groups.

Since the goal of the landmarks is to create the data points for downstream geometric morphometric methods, we tested the consistency of some of the typical GMM statistical parameters (size and mean shapes) across methods. As shown in Table 3, single atlas based annotations performed poorly in all estimated parameters. Improved single atlas showed some progress, but still remained significantly different from GS in all parameters at α < 0.1. The multi-atlas method was statistically indistinguishable from the GS when estimating the mean shape both for Euclidean Distance Matrix Analysis (EDMA) and Generalized Procrustes Analysis (GPA). Visualizations of GPA mean shape estimates for all three comparisons were provided in online supporting documents. Centroid size, the typical size measure used in GMM, was significantly different from the GS for all methods, although the absolute difference is very small, about 0.2 % of the GS centroid size for all methods, to be biologically relevant.

**Table 3:** *P* values from statistical tests of different GM parameter estimates

	EDMA FORM	GPA SHAPE (one sample)	GPA SHAPE (two sample)	Centroid Size	Centroid size R^2^
GS v Atlas	0.010	<0.001	<0.001	<0.001	0.96
GS v Improved Atlas	0.083	0.076	0.091	<0.001	0.97
GS v MAAP	0.476	0.1399	0.157	<0.001	0.95

For EDMA, we used the Form procedure of the WinEDMA (Cole, 2002), which used a permutation test with 100,000 replicates to establish the significance. For GPA we used the testmeanshapes function from R shapes package. A permutation test was used for the one sample test (assuming exchangeability between groups), whereas a bootstrap procedure was used for two-sample test. 50,000 replicates were used in both cases. Because the number of samples were low for a true multivariate test such as Hotelling T^2, we reported the Goodall F-test metric which uses the sum-of-squared Procrustes distances to measure SS (Goodall, 1991). This test is also known as Procrustes ANOVA. A paired *t*-test was used to compare centroid size estimates. All comparisons were run as separate statistical tests. All groups contained the identical set of samples (*N* = 36 per group). Adjusted R^2^ results are from linear regressions of centroid size from automated methods on GS centroid size

## Discussion

We chose DRAMMS as our registration and atlas building platform due to its documented performance in wide variety of imaging datasets and its extensive documentation [26–28]. By no means, it is the only platform to execute atlas based landmark annotation. Investigators have a large variety of tools to choose from; NeuroInformatics Tools and Resources website (https://www.nitrc.org) currently lists 40 such atlas building frameworks. However, careful attention has to be paid on how the algorithms are implemented and the biases that might be associated with each of them.

Based on our tests, MAAP outperforms single atlas methods and performs as well as our GS. Even though the improved single atlas shows promise, we favor the multi-atlas approach due to its flexibility to capture variation. We expect the improved single-atlas methods to perform sufficiently well in datasets like this where there are no clear outliers in morphology. However, as the variation in the study population increase (inclusion of mutants, knock-outs, different strains), performance of the improved atlas may suffer, because the variation in the reference templates is reduced to a mean estimate of landmark location on the atlas. Thus, back projection of this estimate is solely dependent on how well outlier sample registers to the atlas. In MAAP, since each template is registered against the target and a ‘voting mechanism’ such as shape-based averaging (SBA) is used to determine the final location of the landmark, the variation in the reference templates is still presented in the outcome. Therefore, we will focus most of our discussion on MAAP.

Because the similarity between template and target images determines the landmark accuracy, the choice of templates may have an impact on the outcome of automated annotation. In current study, we used the K means clustering that is available through the MAAP package to select our samples that served as templates. K-means clustering requires the investigator to determine the number of templates to be chosen. We chose 10 samples to serve as templates, because we wanted to imitate a situation where a small subset of the study population is used to annotate the remaining samples. This choice, in practice, depends on number of factors such as the variation in the datasets as well as the computational infrastructure available at hand. In our study the phenotypic variation was not extremely large, it is quite possible that most samples would have served successfully as templates, so an argument for choosing the templates in random can be made. However, in larger studies involving knock-outs or mutants, there will be morphological outliers. If the selected templates are enriched in number of outliers due to random sampling, it is possible that less than optimal automated landmark results can be obtained. Our recommendation is that the investigator spends some time to do a preliminary analysis of variation and carefully evaluate the templates. For consistency we used the same set of 10 templates between improved single atlas and multi-atlas methods.

Although an increased number of templates means increased computational time, DRAMMS and the associated multi-atlas consensus algorithm and software can be readily multi-tasked through the use of a grid computing environment where registration of the templates can be run simultaneously. On a single compute node with dual Intel Xeon E5-2690v2 processors (20 physical cores), on average a single MAAP sample took 105 minutes (+/−6 min) when 10 templates were used to landmark targets. For every MAAP target, all 10 templates contributed to the landmark calculation. Volumes were approximately 200x600x300 voxels in size. This execution time corresponds to approximately 27 samples in 24 h, assuming two target jobs can be submitted simultaneously to utilize all available cores. Using only two compute nodes, a throughput of more than 50 samples per day can be achieved for this dataset. Addition of more templates, or working with larger datasets (such as skulls) will increase the computation time, but this can be easily offset by using additional compute nodes in tandem. It should be noted that about 2/3rd of the computational time was spent on registering the templates to the target, which is trivially parallel. The remaining 1/3rd of the computation time (~37 min) was spent on the label fusion through shape-based averaging (SBA) to calculate the final location of landmarks on the target. This is a serial task and cannot be parallelized. Apart from SBA, there are multiple label fusion algorithms, such as majority vote (MV) and simultaneous truth and performance level estimation (STAPLE). MV is the simplest label fusion algorithm, in which the mode of the possible values is for the selection.  Unfortunately, if there is not sufficient overlap of the warped label maps, the algorithm will not yield any result making it a poor choice for study population with a large degree of variance. STAPLE is an expectation-maximization algorithm that iteratively estimates the true segmentation from the raters’ performance and the raters’ performance (sensitivity and specificity) from the true segmentation estimate. STAPLE has been shown to outperform MV and SBA when labels maps are quite dissimilar. Our mandible dataset contains only a subtle amount of variation. After deformable registration the average dice image similarity score is approximately 0.99. Therefore, the warped labels are very similar and different label fusion methods yield nearly identical results. This is demonstrated by comparing STAPLE, SBA and MV label fusion on the same template set (Additional file 1: Figure S1).

For datasets with natural populations or mutants, investigators may benefit from more state-of-the-art label fusion methods such as Spatial STAPLE and COnsensus Level, Labeler Accuracy, and Truth Estimation (COLLATE). Spatial STAPLE improves upon STAPLE by adding a voxelwise performance level field that is unique to each rater, improving local sensitivity [29]. COLLATE label fusion focuses on the notion that some regions, such a boundary, are more difficult to segment while other regions, near the center of large label or high contrasts edges, are intrinsically easy to label [30]. For this particular study either method is unlikely to appreciably improve the results, which are already in very good agreement.

### Reliability of estimated shape parameters

Although the average linear distance between the corresponding landmark pairs are small in this study (Fig. 2, Table 2), it is still possible that they may differ in a systematic way which can impact the covariation between landmarks. This in return will impact any analysis that uses the variance-covariance (VCV) matrix to derive secondary shape variables (such as principal component scores) that are typically tested against environmental and genetic factors. This would be particularly troublesome if expert and automated annotations are mixed in the statistical analysis. To the best of our knowledge no other study that focused on the fully automated or semi-automated annotation of landmarks has investigated the similarity of the VCV matrices or any of the GMM parameters, such as mean shape, from different methods.

The very small, but highly significant difference in the centroid size suggests to us that there might be a systematic bias in this dataset. We used Box M test to test for the homogeneity of covariance matrices estimated from GS and MAAP. To make sure that the covariance patterns do not influence each other due to joint superimposition, we conducted separate GPA analyses. Because of the low number of samples (36) *versus* the number of coordinate variables (48), we opted to use principal component scores instead of Procrustes residuals. We chose principle components (PCs) that expressed 1 % or more of the variation, which resulted in selecting the first 15 PCs accounting for 88 % of the shape variation in both datasets. Based on this dataset, we failed to reject the null hypothesis that the covariance matrices for GS and MAAP are equal (Chi-square = 141.78 df = 120, *p*-value = 0.08512). Due to high intraobserver error associated with some of the landmarks (eg. 10), this may not be a particularly suitable dataset to investigate the issue. We advise that further investigation should be conducted for any automated or semi-automated method for the consistency of parameters estimated from manual annotations and corresponding methods. Until a clearer understanding of consistency of VCV estimates from manual landmarks and any automated method emerges, we advise against mixing samples that were obtained by different annotation techniques. We do not see this as a serious drawback, as the proposed MAAP approach is intended for experiments with large sample sizes where a small portion of the population can be set aside to serve as templates.

### MAAP *versus* TINA

The MAAP method is comparable to a recently published semi-automated landmark annotation technique by Bromiley et al. [24] as developed for the TINA Geometric Morphometrics toolkit (TINA). Similar to MAAP, TINA involves the registration of the template images to the target image and then transferring the landmarks to the target images. The main difference between these two approaches is the choice of registration procedures. TINA uses a course to fine resolution iterative affine registration paradigm; where local regions of interest (ROI) of decreasing size are defined around each landmark. MAAP, on the other hand, uses a global deformable registration. Another difference is the use of array based voting method based on the image noise to determine the final landmark location from the location of the registered template images in TINA. However, because of the conceptual similarity between approaches, we evaluated the performance of MAAP against TINA. Using the same 10 templates from MAAP, we constructed a template database in TINA. 36 samples automatically annotated using this database. We chose LMs 3, 4, 5, and 6 to do the initial global registration. All other settings were left as default.

**Fig. 3:**
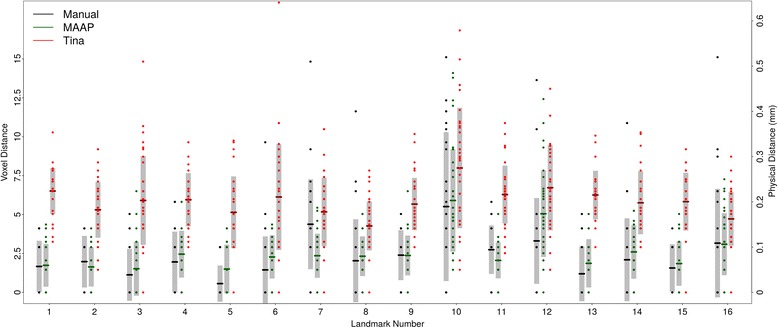
Comparison of MAAP and TINA results with respect to gold standard. Conventions same as Fig. 2. Because TINA reports values only as integers, our results from Fig. 2 were also rounded to the closest integer

Thanks to affine only registration, TINA is extremely fast. On the same computer hardware, it took only 39 minutes to process 36 samples, averaging 1.1 min/sample, two orders of magnitudes faster than MAAP. This, however, came at the expense of landmarking accuracy. Every landmark performed poorly with TINA compared to MAAP. Mean errors associated with TINA were 1.3 to 5.5 times larger than MAAP (Fig. 3).

**Fig. 4:**
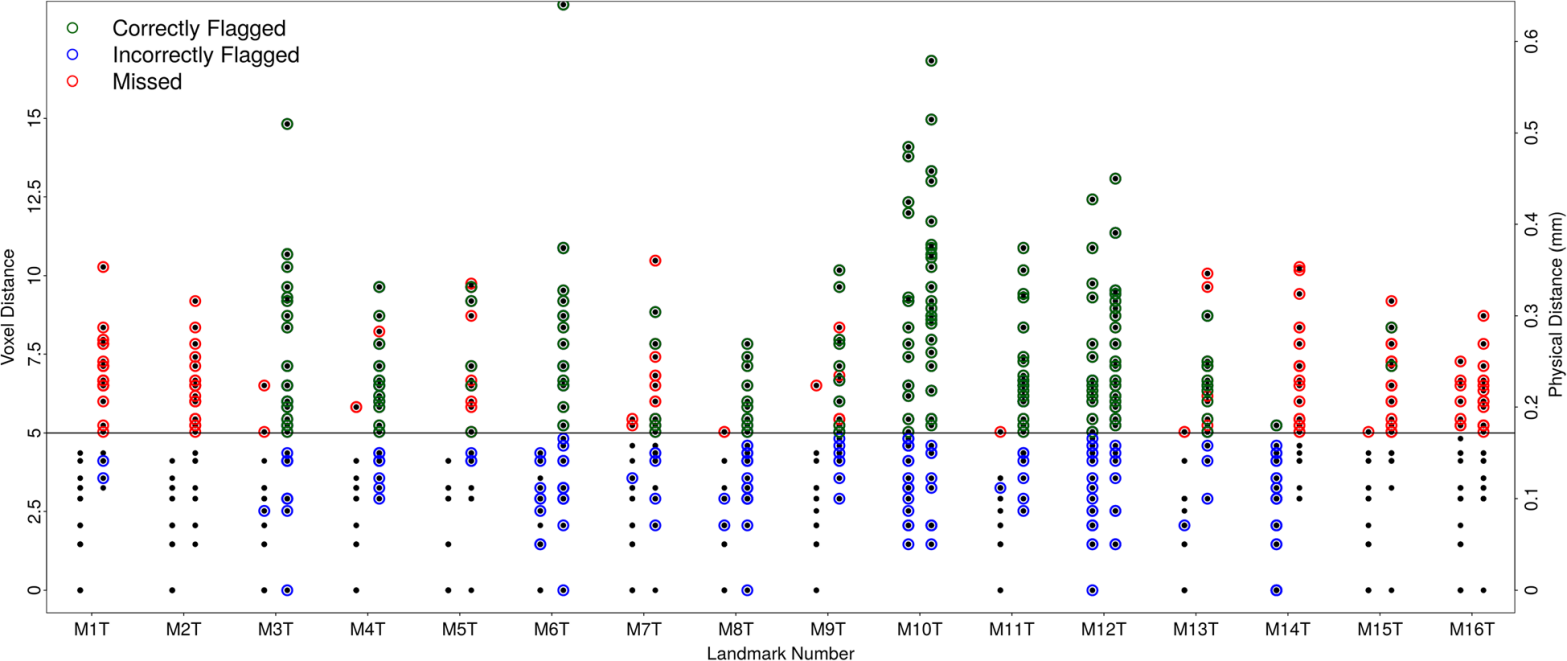
Comparison of the outlier detection performance in MAAP and TINA. For each landmark left column (M) is the result for MAAP and right column (T) is the result for TINA. Each data point represents the difference of the estimated landmark to the corresponding GS one. Horizontal line at five voxel mark represent the threshold specified to assess the outliers in both methods. For MAAP, if two or more of the templates (out of 10) were outside of this threshold range, the software flagged the landmark for manual verification. Green circle indicates landmarks that are correctly flagged as outliers, red circle indicates landmarks that are in reality outliers but missed by detection software, and blue indicates landmarks that were incorrectly flagged since they were below threshold

Error detection is an important quality control tool for a large scale landmarking study. We used a user specified threshold to evaluate the distance between each landmark location given by each template and its final location. If any two distances (out of 10 estimates) exceeds the specified threshold, the landmark is flagged as potentially problematic. We tested our approach on our data using a five voxel (0.172 mm) threshold. A total of 120 out of 576 landmarks were flagged as potentially problematic (Fig. 4). Of the landmarks whose error from the GS was actually greater than the preset threshold, only 6.7 % were not identified by the algorithm. Conversely, our algorithm also selected a large number, 88 of out 120, landmarks that were flagged as problematic, yet were below the specified threshold when compared to the GS. In these cases, averaging the landmark locations yielded a good result even if there were more than two outliers in template landmark locations. Obviously, it is not possible to automatically identify these ‘false hits’ without the GS. In real world application the user still needs to visualize the LMs and visually confirm. Because there is only one estimate of landmark location in single atlas based methods, this kind of outlier detection mechanism cannot be implemented in that framework.

We also evaluated the performance of our outlier detection against TINA’s outlier detection tool, using the same five voxel threshold to flag outliers (Fig. 4). TINA performed quite poorly, with very high rates of missed LMs (1, 2, 16), as well as false positives (3, 4, 6, and 8). LMs 10, 12, and 14 were challenging for both methods due to the high number of missed and incorrectly flagged LMs. The performance of TINA’s outlier detection tool was somewhat surprising because of the previously reported false positive rate of 0.5 % (Bromiley et al., 2014). The outlier detection in TINA was only tested in terms of detecting points that were in completely the wrong place (i.e. digitization errors, or LMs out of sequence). It is based on the spread of predictions in the voting arrays, and relies on outliers being well-separated from all other data. The thresholds are typically set around 2.5 sigma of the distribution in the array (Bromiley et al. 2014). Our study population is phenotypically similar in shape, and less variable than the natural mouse population that Bromiley et al. (2014) used to test TINA. This causes the votes to form a single distribution with no distinct outside peaks. We tried TINA with different sigma values (results not shown), however, it appears there is no optimal parameter setting that would give a good classification.

It appears that TINA is far faster than MAAP at the expense of LM accuracy for this particular dataset. The tradeoff between speed and quality is difficult to quantify, and will likely depend on a number of factors such as the number of landmarks to be revised, availability of man-power to do the correction vs the availability of high-performance computing environment, and likelihood of human error. Given that on average it takes about a minute to manually annotate a landmark (Bromiley et al., 204), MAAP is slower than doing the annotations manually. But it should be noted that the majority of the computational time was spent on conducting the deformable global registration. The number of landmarks to be annotated has no bearing on speed of the registration which is solely dependent on the size and similarity of datasets. As the number of landmarks in the dataset increases significantly, we expect MAAP to be rather competitive.

In the future we plan to investigate the metrics associated with the underlying label fusion algorithms, both SBA and simultaneous truth and performance level estimation (STAPLE), to develop a more robust outlier detection tool that will avoid the false hits, thereby reducing the user intervention time. STAPLE seems particularly promising, because it considers a collection of segmentations and calculates a probabilistic estimate of the true segmentation. The estimate of the true segmentation is computed by estimating the optimal consolidation of the segmentations, weighting each segmentation by an estimated performance level together with constraints for spatial homogeneity. Such estimated performance weights from STAPLE [31], or distance map measures from SBA [32] could be used in place of a local registration quality measure. Another possibility is to look at the dispersion of estimated landmarks around the centroid (the best estimate) and rank them by their eigenvalues or a minimum covariance determinate estimator. Also, more research is needed to determine the performance of registration algorithms (affine vs deformable) and their spatial domains (local *versus* global) in context of datasets that display wide morphological variation in all landmarks, such as developmental datasets.

## Conclusions

In summary, the proposed MAAP framework as implemented through DRAMMS for landmarking shows promise as an effective procedure for accurately annotating large datasets that are typical of large phenotyping or genetic mapping studies. Although MAAP is slower compared to other alternative, TINA, its performance in accuracy is far better than TINA, both in terms of approximating the manual landmarking, as well as detecting potentially erroneous LMs.

With a more robust outlier detection method, MAAP has the power to facilitate high-throughput analysis of large datasets through use of high performance computing environments. It provides a flexible framework necessitating no mathematical or geometric definition of anatomical structures. It enables to channel investigators valuable time to improve the precision of the annotated templates, thus reduce the potential for human error.

## Methods

### Dataset and imaging

The current study population is a subset of our unpublished dataset consisting of mandibles from a mixed population of C57BL/6 J mice there were chronically exposed to different dosages of ethanol solution *in*-*utero*. Fifteen animals were born to mothers (*N* = 3) that obligatorily consumed 10 % ethanol/water mixture throughout their pregnancy. Ten animals were born to mothers (*N* = 2) that obligatorily consumed 15 % ethanol/water mixture for the first eight days of their pregnancy. Thirty animals were born to mothers (*N* = 5) that consumed only water. Litters from all mothers were euthanized at P75 and their heads were scanned with Skyscan 1076 microCT scanner (Skycan, Co) using a standardized acquisition settings (0.5 mm Al filter, 55 kV, 180uA, 80 ms exposure, 0.7° degree rotations). Three images were obtained and averaged at each rotation. Grayscale image stacks were reconstructed at 34.42 micron using identical settings for all samples. All animal procedures used in this study were approved by the Institutional Animal Care and Use Committee of the Seattle Children’s Research Institute.

### Manual landmarking

**Fig. 5:**
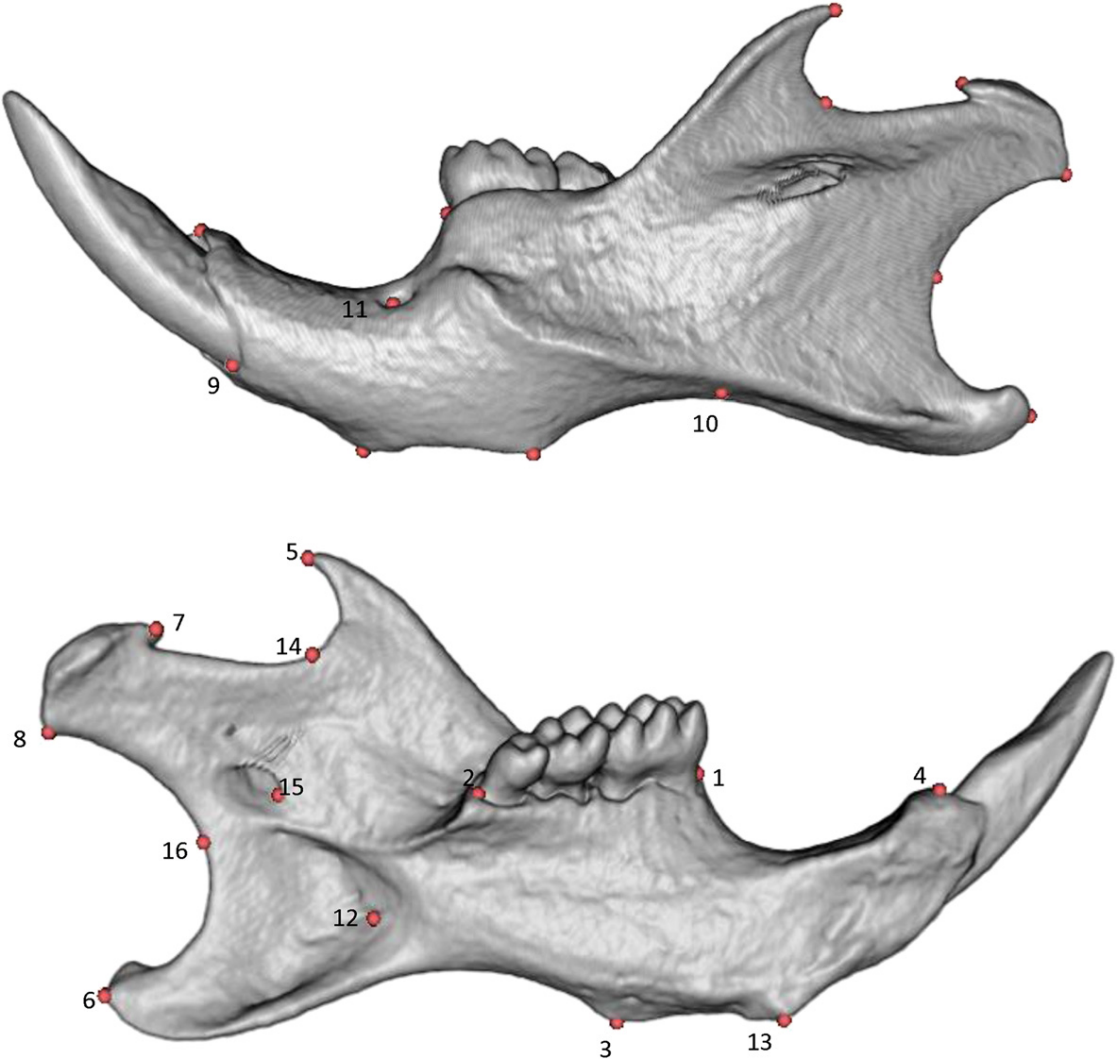
Landmarks used in the study. Further information on landmarks definitions were provided as an online supporting document

Following image reconstruction, a trained technician segmented the left and right hemi-mandibles from the skull using Ctan (Skyscan Co). Segmented left hemi-mandibles were imported into 3D-Slicer and visualized using a fixed rendering and threshold setting. The technician was initially trained on 16 mandibular landmarks (Fig. 5) compiled from literature [19, 33, 34]. The training dataset consisted of a set of C57BL/6 J mandibles that were of the identical age, but that were not part of this study to avoid any ‘learner’s bias’ (i.e. shifting the landmark positions gradually after getting exposed to more phenotypic variation, or learning the software better). After the training, the technician annotated all samples in the study twice with four weeks separating the first and second attempts. Only overt digitization errors (such as mislabeled landmarks) were corrected and no further refinement of the landmarking process was performed. Median and mean error in corresponding landmarks between two manual digitization attempts were 0.048 mm (1.4 voxels) and 0.073 mm (2.1 voxels) respectively. Some landmarks (e.g. 10 and 16) had higher digitization errors associated with them (Table 2). These are typical of manually annotated type III landmarks (e.g. deepest point on a curvature). We averaged these two sets and designate it as our GS for the purpose of this study.

### Atlas building overview

We used the open source DRAMMS deformable registration software and atlas building pipeline in this study [35]. The DRAMMS registration software consists of two major components, attribute matching and mutual-saliency weighting. Attribute matching characterizes each voxel by a high dimensional vector of multi-scale and multi-orientation Gabor attributes. This method provides more information than the traditionally used intensity information [26, 28, 36, 37]. Because the reliability and accessibly of correspondence varies across anatomical structures, mutual-saliency upweights regions of the volume where correspondence can be reliably established between the source and target images. The preferred use of reliable correspondence reduces the negative impact of outlier regions on the registration quality [26, 28, 36, 37].

Atlas construction was performed by using the DRAMMS deformable registration in a classic unbiased population-registration framework [38]. The atlas construction framework iteratively finds a virtual space that resides in the centroid of the study population (centroid meaning that the deformations needed to transform all subjects into this virtual space sum up to zero everywhere in this virtual space). Therefore, the constructed atlas is unbiased to any subject in the population, and is hence representative of the mean anatomy/geometry of the population [25, 38].

### Single atlas annotation

Atlas based landmarking is the process of transferring landmarks from an atlas to the individual samples in the population. As the atlas resides in the center of the deformation space it minimizes the average deformation magnitude, thus providing the best population wide registrations. The annotation process is initiated by building an atlas using the whole population or a representative subset of the population, which is later manually annotated with landmarks by an expert. Once created, these landmarks are first converted to spheres with a small radius (4 voxels) and then back-projected to the individual samples by reversing the transformation. However, unlike the individual samples that constitute it, the surface selection on an atlas is non-trivial. A surface, generally bone (or other tissue of interest), is typically defined *via* a set voxel threshold that is specific to the density of the structure. However, because the atlas is a constructed dataset, grayscale values of the voxels do not necessarily correspond to density of the tissue of interest (mandibular bone in this case). Thus, a threshold may not consistently represent bone or tissue values as it does in a single microCT image. We explored effects of using different criteria for selecting the surface to annotate as well as the effect of the initializing sample on the outcome of the atlas.

The same technician landmarked the final chosen surface from the previous steps in three different attempts. We used the average of the three landmark sets as the best estimate of the landmark locations on atlas. A label map, in which each landmark was represented using a spherical label with a radius of two voxels centered on the landmark, was created. This label map was projected back to the original samples by reversing the transform and final coordinates of the landmarks were estimates.

### Improved single atlas annotation

Due to the arbitrariness of selecting the surface on atlas, we explored the option of using a subset of manual annotations to estimate the positions of the landmarks on atlas. Landmarks from these samples were warped onto the atlas, and averaged to provide an estimate of landmarked atlas. Finally, this set of averaged LMs was projected back to the remaining samples, similar to the single atlas annotation.

### Multi-atlas annotation procedure (MAAP)

In this approach, a subset of the expert annotations was used to annotate the remaining samples also. However, unlike the improved atlas method, they were not reduced to a single best estimate, but all of them contribute to the final estimate to varying degrees. The multi-atlas framework consists of three main modules; template selection, registration, and averaging. The annotation process is based on the multi-atlas segmentation program (hereafter referred as MAAP) that is built upon the DRAMMS registration library [39]. We used K-means clustering on the study population and identified ten samples to be used as templates. Template selection through clustering seeks to find a template set representative of the variation within the sample population. This decrease landmarking bias by minimizing the amount of phenotypic correlation within the template population. Clustering was performed on the vectors of image voxel values, and sought to minimize the within cluster Euclidean distance between members and the mean. The sample closest to the mean of each cluster was selected as a template.

Similar to the single atlas methods each landmark was represented by spherical labels centered on the landmark. One distinct label map was created for each template. It might be beneficial to vary the spherical radius across landmarks based on the observed variation, but this option was not explored. A spatially adaptive label fusion algorithm, shape based averaging (SBA), was used to ensure smooth landmark labels [32]. Templates were automatically selected for label fusion based on the correlation coefficient between registered and target images to mitigate the effect of outliers and poor registrations. Once the warped landmark maps have been averaged, the centroid of each label was taken as the final landmark location.

Since last two methods removed 10 samples from the study population, they were also removed from the single atlas method for the sake of consistency. Four samples were cut in the mandibular symphysis during the initial scan, which affected the registrations. We removed those samples as well. The final sample size used in all figures and statistical tests were 36.

### Comparisons and statistical analysis

We evaluated the performance of each procedure by calculating the linear distance between the corresponding landmarks in automated method *versus* our GS. In return, this difference was compared to the observed human variation in that landmark between the two attempts of manual digitization.

We tested the accuracy of the mean shape estimates of automated methods against the GS both using Generalized Procrustes Analysis (GPA) and Euclidean Distance Matrix Analysis (EDMA), the two most commonly used geometric morphometric methods [10, 11]. Configurations of landmarks are first superimposed on their respective centroids, scaled to unit size, and rotated until the difference between the landmark configurations are minimized through least-squares optimization [10]. In EDMA, all possible paired landmark distances are calculated from the landmark coordinates and converted into a Form Matrix which is expressed as a ratio of two populations, in this case GS and MAAP results [40]. A bootstrap resampling method establishes the confidence intervals associated with each paired landmark distance [40]. We used the Goodall F-test (Procrustes Anova) to assess the difference between mean shape estimates in GPA [41]. All statistical analyses for GPA were conducted in R 3.1.2 [42] using the relevant geometric morphometric packages, shapes and Morpho [43, 44]. We used winEDMA (Cole, 2002) to test for EDMA mean shape differences using its form procedure.

### Availability of supporting data

The data sets supporting the results of this article and its additional files are included within the article.

## Additional file

Additional file 1: Figure S1. Comparison of performance of the three label fusion algorithms discussed in text. **SBA**: Shaped Based Averaging, **STAPLE**: Simultaneous truth and performance level estimation. Results in the main text were based on SBA. However, in datasets like this one, where there are no distinctmorphological outliers, majority vote (MV) performs rather competitively. It is also quite fast; on average it took 1.5 minutes per sample as oppose to 37 minutes SBA took. All three methods performed almost identically. (TIF 471 kb)

## Abbreviations

CF: craniofacial
COLLATE: consensus level, labeler accuracy, and truth estimation
DRAMMS: deformable registration *via* attribute matching and mutual-saliency weighting
EDMA: euclidean distance matrix analysis
GMM: geometric morphometric methods
GPA: generalized procrustes analysis
GS: gold standard
LM: landmark
MAAP: multi-atlas annotation procedure
microCT: micro computed tomography
MV: majority vote
PC: principal component
SBA: shape based averaging
STAPLE: simultaneous truth and performance level estimation
VCV: variance-covariance
