# Supplementary figures and images for: Performance of single and multi-atlas based automated landmarking methods compared to expert annotations in volumetric microCT datasets of mouse mandibles
Source: Front Zool. 2015 Dec 1;12:33. doi: 10.1186/s12983-015-0127-8 (PMC4666065; doi:10.1186/s12983-015-0127-8)

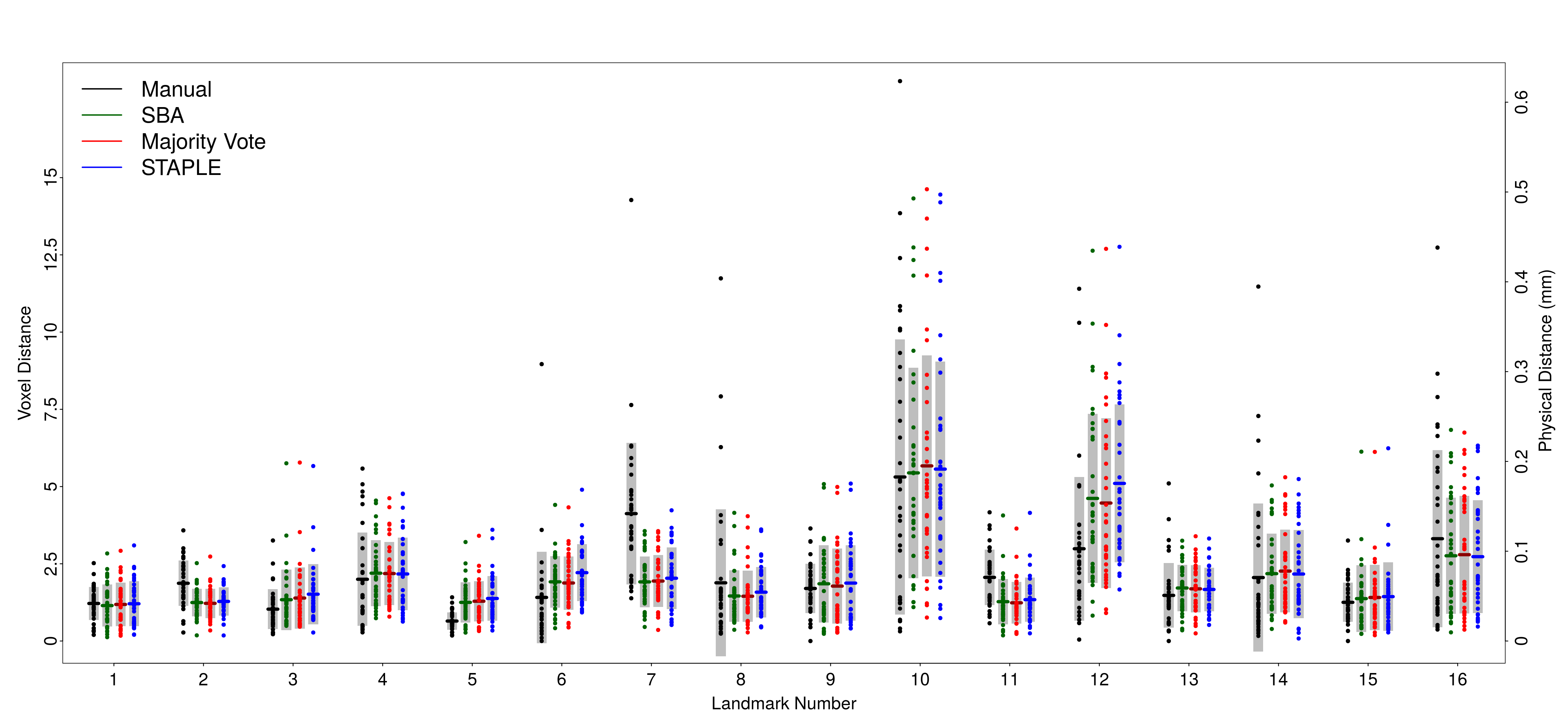

Supplement: Additional file 1: Figure S1. — Comparison of performance of the three label fusion algorithms discussed in text. SBA: Shaped Based Averaging, STAPLE: Simultaneous truth and performance level estimation. Results in the main text were based on SBA. However, in datasets like this one, where there are no distinctmorphological outliers, majority vote (MV) performs rather competitively. It is also quite fast; on average it took 1.5 minutes per sample as oppose to 37 minutes SBA took. All three methods performed almost identically. (TIF 471 kb) [file 12983_2015_127_MOESM1_ESM.tif]
